# Face Liveness Detection Using Defocus

**DOI:** 10.3390/s150101537

**Published:** 2015-01-14

**Authors:** Sooyeon Kim, Yuseok Ban, Sangyoun Lee

**Affiliations:** Department of Electrical and Electronic Engineering, Yonsei University, 134 Shinchon-dong, Seodaemun-gu, Seoul 120-749, Korea; E-Mails: sykim1221@yonsei.ac.kr (S.K.); van@yonsei.ac.kr (Y.B.)

**Keywords:** face liveness detection, anti-spoofing, defocus, 2D fake face, webcam

## Abstract

In order to develop security systems for identity authentication, face recognition (FR) technology has been applied. One of the main problems of applying FR technology is that the systems are especially vulnerable to attacks with spoofing faces (e.g., 2D pictures). To defend from these attacks and to enhance the reliability of FR systems, many anti-spoofing approaches have been recently developed. In this paper, we propose a method for face liveness detection using the effect of defocus. From two images sequentially taken at different focuses, three features, focus, power histogram and gradient location and orientation histogram (GLOH), are extracted. Afterwards, we detect forged faces through the feature-level fusion approach. For reliable performance verification, we develop two databases with a handheld digital camera and a webcam. The proposed method achieves a 3.29% half total error rate (HTER) at a given depth of field (DoF) and can be extended to camera-equipped devices, like smartphones.

## 1. Introduction

At present, many people deal with personal business using portable devices. From unlocking cellular phones to financial business transactions, people can easily conduct their individual business tasks through such a device. Due to this trend, personal authentication has become a significant issue [1]. Instead of using a simple PIN code, industries have developed stronger security systems with biometric authorization technology [2]. Biometric traits, such as face, iris and fingerprint, are very powerful factors to protect one's private information.

However, attempts to invade security systems and steal personal information have been increasing. One type of these attacks involves using fake identities. Spoofing faces and fingerprints are threatening security systems and privacy. This would not matter if current face recognition (FR) systems were secure, but current systems cannot distinguish fake faces from real faces. In some cases, the FR system embedded in cellular phones gives approvals to forged faces. This phenomenon is an example of weakness in the biometric system. If this problem remains unsolved, anyone will be able to easily obtain others' personal information in order to commit identity-related crimes. For this reason, technological defense against spoofing attacks is necessary, so as to protect personal systems and users' private data. Over the last decade, researchers have shown steady progress in developing anti-spoofing technologies [3]. Most of these methods concentrate on exploiting features obtained from the analysis of textures, spectrums and motion in order to detect face liveness.

In this paper, we propose a new method to secure face identification systems from forged 2D photos. The key factor of our methods is that we utilize the camera function, variable focusing. In shape-from-focus, it is possible to construct 3D images using focus measures [4,5]. Even though we need not recover the 3D depth images, we use the characteristics of the defocusing technique in order to predict the existence of the depth information. By adjusting the focusing parameters, parts of the image that are not in focus become blurry. With this function, we can evaluate differences in the degree of focus between real faces and fake faces and use this information to detect face liveness. To evaluate our method, we organized two databases using a handheld digital camera and a webcam.

The remainder of this paper is organized as follows. In Section 2, we discuss previous studies on face liveness detection and the theoretical background of camera focusing. Our proposed methodologies are stated in Section 3. In Section 4, experimental results are shown and the details are discussed. Finally, concluding remarks are provided in Section 5.

## 2. Related Work

### 2.1. Countermeasures against Spoofing Faces

Numerous approaches to minimize vulnerability to attacks using spoofing faces have been proposed. In early research, intrusive methods that request user cooperation, such as speaking phrases and shaking one's head [6], were developed. However, these approaches cause users inconvenience and rely on users' cooperation. For this reason, many researchers have attempted to develop non-intrusive methods.

Depending on the type of attack, methods can be categorized into three groups: 2D static attacks (facial photographs), 2D dynamic attacks (videos) and 3D attacks (masks). Skills and devices for disguising one's identity have evolved gradually. Masks and videos are examples of advanced spoof attacks. Some studies have focused on protecting FR systems from these advanced attacks [7,8]. However, due to the difficulty and cost of obtaining such advanced tools, 2D static attacks, such as photographs, have been widely used by attackers. In this chapter, we review studies for detecting 2D facial photo-based spoof attacks.

There are three main spoof detection approaches, depending on the characteristics of input faces. The first approach is based on textures. Real and fake faces have different texture characteristics. Some studies have used texture to detect forged faces. Kim et al. [9] applied local binary patterns (LBP) for texture analysis and power spectrum for frequency analysis. Määttä *et al.* [10] and Bai *et al.* [11] also detected face liveness by examining micro texture with multiscale LBP. Peixoto *et al.* [12] proposed a method to detect and maintain edges (high-middle frequencies) with different Gaussian characteristics under poor illumination conditions. In [13], the authors extracted essential information for discrimination using a Lambertian model. Singh *et al.* [14] proposed a method to classify real faces based on a second-order gradient. This approach focuses on differences between skin surfaces of real and fake faces. Kant *et al.* [15] presented a real-time solution using the skin elasticity of the human face. Approaches with a single image have advantages in terms of low capacity and simplicity.

The second approach uses motion information. Signs of liveness, such as eye blink and head movements, are clues to distinguish motionless spoofing faces. Image sequences can be used to perceive movements. These factors are exploited intuitively [16–20]. In addition, optical flow and various illumination approaches are helpful to analyze the differences between real and fake faces [21–25]. Applying the entropies of RGB color spaces is one factor in face liveness detection [26]. To make a robust system, several methods use a combination of static and dynamic images [18,27].

The last approach is based on 3D facial information. The obvious difference between a real face and a fake face is the presence or absence of depth information. Human faces have curves, while photos are flat. By considering this feature, researchers have classified spoofing attacks. Wang *et al.* [28] suggested an approach to detect face liveness by recovering sparse 3D facial models, and Lagorio *et al.* [29] presented a solution based on 3D facial shape analysis.

### 2.2. Background Related to Focusing

**Figure 1.**
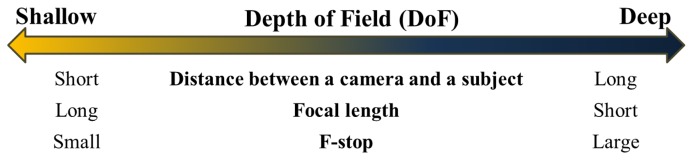
Factors for the adjustment of the depth of field (DoF).

Unlike previous research, our method utilizes the effect of defocus. Defocusing is exploited to estimate the depth in an image [4,5,30]. The degree of focus is determined by the depth of field (DoF), the range between the nearest and farthest objects in a given focal plane. Entities in the DoF are perceived to be sharp. In order to emphasize the effect of defocus, the DoF should be narrow. There are three parameters that modulate DoF, and Figure 1 shows those conditions for shallow DoF [31]. The first factor is the distance between the camera and the subject; a short distance produces a shallow DoF. The second factor is the focal length, which is adjusted to be longer for a shallow DoF. The last factor is the lens aperture of the camera, which is made wider to produce a shallow DoF. Using these options, we can achieve images with a narrow DoF and a large variation in focus [31].

### 2.3. Previous Work with Variable Focusing [32]

In the previous work [32], a method for face liveness detection using variable focusing was suggested. Two images sequentially taken at different focuses are used as input images, and focus features are extracted. The focus feature is based on the variation of the sum modified Laplacian (SML) [33] that represents the degrees of focusing. With the focus feature and a simple classifier, fake faces are detected. 2D printed photos are used as spoofing attacks, and a database composed of images with various focuses is produced for evaluation. When DoF is shallow enough to make the only partial area blurred, this method shows good results. However, at a deep DoF, the performance is deteriorated. In order to make up for the weakness of the previous work, we propose an improved method in this paper. Extracting local feature descriptors and frequency characteristics, as well as the focus feature from the defocused images, we detect spoofing faces. Moreover, the quantity of the database is increased, and various experiments are performed to achieve the best result. A detailed explanation will be described in the following sections.

## 3. Proposed Methodology

**Figure 2.**
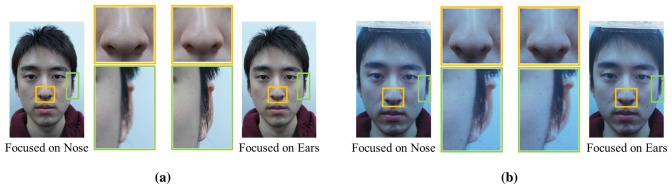
Partially focused images of (**a**) real faces and (**b**) fake faces.

In this section, we introduce new FR anti-spoofing methods using defocusing techniques. From partially defocused images, we extract features and classify fake faces. The most significant difference between real and fake faces is the existence of depth information. Real faces have three dimensions, with the nose and ears being relatively far from each other. This distance can be used to adequately represent the depth information. Depending on the object or place of focus, the ear area might or might not be clear, as shown in Figure 2a. Unlike real faces, 2D spoofing faces are flat. There is little difference in clarity, regardless of the focus (Figure 2b). We emphasize this characteristic in order to discriminate real faces from 2D faces.

In order to maximize the effect of defocus, we must adjust the DoF to be shallow, as mentioned in Section 2. However, according to the type of camera, the adjustment of DoF may not be possible. Therefore, we obtain input images using two cameras, a handheld digital camera and a webcam. We will explain image acquisition in the following section.

**Figure 3.**
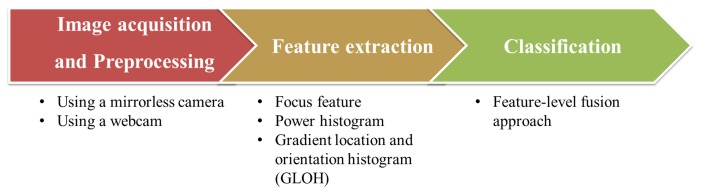
Flowchart of face liveness detection using defocus.

Our system is composed of three steps: image acquisition and preprocessing, feature extraction and classification (Figure 3).

### 3.1. Image Acquisition and Preprocessing

In our method, image acquisition is an important factor in performance. As mentioned in the previous section, a narrow DoF increases the effect of defocus and assists with detecting fake faces. However, not every camera can easily change its DoF and focal plane. If people use handheld digital cameras, such as DSLR (digital single lens reflex) and mirrorless cameras, the DoF can be made shallow by directly controlling camera settings and the areas of desired focus can be manually selected. However, when users utilize webcams and cameras embedded in cellular phones, they cannot accurately manipulate the DoF. Moreover, the position of the focal plane is inexact with such cameras. Therefore, the process of image acquisition needs to vary with the type of camera. We will introduce two methods appropriate for a handheld digital camera and a webcam, respectively.

#### 3.1.1. Using a Handheld Digital Camera

With handheld digital cameras (DSLR camera, mirrorless camera, compact digital camera, etc.), it is possible to manually control the focal plane and DoF. Hence, two sequential focused facial images are obtained for use in these experiments: one is focused on a nose and the other on ears (Figure 2). When we set the focus on the ears and nose, we can tap on the LCD panel or turn a focus ring in accordance with the type of handheld digital camera. In this paper, a mirrorless camera (SONY-NEX5) is used, and it has a focus ring. Therefore, we acquire the focused images, turning the focus ring and checking the sharpness in the regions of the ears and nose with our eyes.

**Figure 4.**
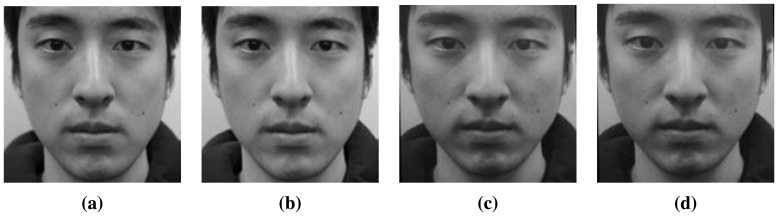
Real face images focused on (**a**) the nose and (**b**) the ear; and fake face images focused on (**c**) the nose and (**d**) the ear.

In the preprocessing step, we geometrically normalize images based on the location of the eyes [34]. In every image, the positions of faces are slightly different. For accurate comparison, faces must be aligned. Based on the coordinates of the eyes, we translate, rotate and crop facial images. The eyes can be automatically detected by using feature templates. In this paper, however, we select the correct positions of the eyes manually in every image and save the coordinates. Figure 4 shows the normalized images produced in the present study. Figure 4a,c is focused on the nose (I*_N_*) and Figure 4b,d on the ears (I*_E_*).

#### 3.1.2. Using a Webcam

The focus in a webcam is controlled by adjusting the plastic lens in and out. However, the DoF is unknown, and it is difficult to select the focus area without the use of a supplemental program. Therefore, unless the program is used, it is not easy to obtain images focused on either the nose or ears. In order to acquire input images with a webcam, we approach the problem in a different way.

**Figure 5.**
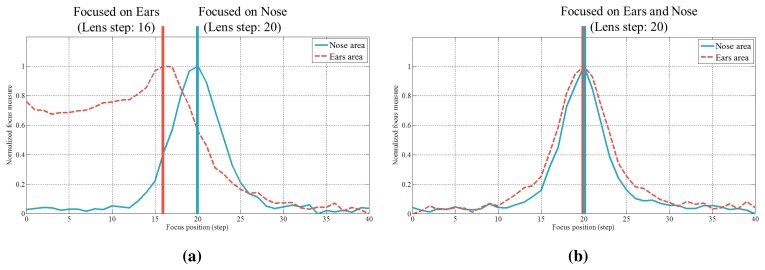
Variations of focus measures in accordance with lens steps ((**a**) real face and (**b**) fake face).

**Figure 6.**
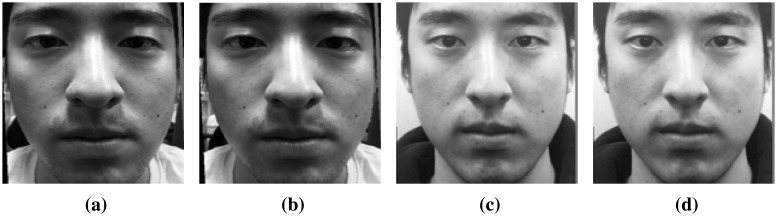
Normalized webcam images (real face images focused on (**a**) the nose and (**b**) the ear; and fake face images focused on (**c**) the nose and (**d**) the ear).

Although it is not possible to accurately take images focused on either the nose or ears when using a webcam, it is possible to obtain image sequences by changing the lens motor step. Depending on the adjustment of the lens, the focal plane varies, producing images with different focal planes. From the image sequence collected here, we select two images, I*_N_* and I*_E_*. I*_N_* and I*_E_* denote the normalized images for which the nose and ear area are in focus, respectively. In order to determine these images, we detect the nose and ears and calculate the degrees of focus in those areas [4]. As mentioned before, the centers of the eyes and the regions of the ears and nose are selected manually in this paper. When the value of a specific area is at a maximum at the *k*-step, that region is in focus. Figure 5 depicts the changes in focus values in accordance with the lens step. In Figure 5a, the nose area is in focus at the 20th step and the ears area at the 16th step. With fake faces, the steps of the maximum focus values for the nose and ears are same, as shown in Figure 5b. This allows one to distinguish between real and fake faces. Through this procedure and normalization, we can choose two images as I*_N_* and I*_E_* (Figure 6).

### 3.2. Feature Extraction

To detect forged faces, features are extracted from normalized images. In this paper, we use three feature descriptors: focus, power histogram and gradient location and orientation histogram (GLOH) [35].

#### 3.2.1. Focus Feature

**Figure 7.**
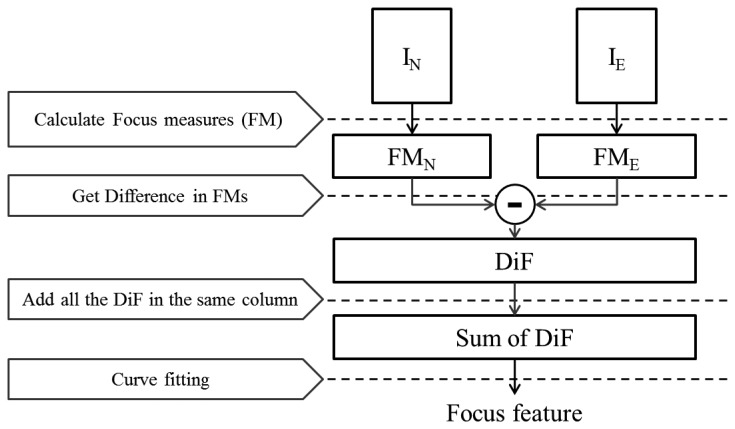
Flowchart of focus feature extraction.

The focus feature is related to the degree of focusing. In the previous study [32], this feature was suggested and used for classifying fake faces. Figure 7 shows the flowchart for extracting focus features.

Using several focus measures [4], we can numerically calculate the focus levels in each pixel. There are various focus measures, such as Laplacian-based measures and gradient-based measures. We will show the performance in accordance with the focus measures.

**Figure 8.**
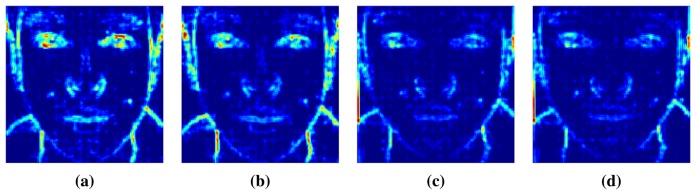
Modified Laplacians (LAPMs) of real face images focused on (**a**) the nose and (**b**) the ear, and LAPMs of fake face images focused on (**c**) the nose and (**d**) the ear.

The images in Figure 8 are the results of modified Laplacian (LAPM) focus measure calculations. LAPM is one of the focus measures introduced in [4,33]. This is presented as the sum of transformed Laplacian filters. Figure 8a,b shows the LAPMs of a real facial image focused on the nose and ears, and Figure 8c,d shows the LAPMs of a fake facial image focused on the nose and ears. We denote the LAPM of nose-focused images by LAPM*_N_* and the LAPM of ear-focused images by LAPM*_E_*. In LAPM*_N_* and LAPM*_E_*, bright pixels represent high values of LAPM, and those regions are in focus with sharp edges. On the contrary, out-of-focus regions have severe blurring, lose edge information and have low values of LAPM. In the case of real faces, the nose area in LAPM*_N_* (Figure 8a) is brighter than that in LAPM*_E_* (Figure 8b). However, there is little difference between the LAPM*_N_* and LAPM*_E_* of fake faces (Figure 8c,d). Consequently, by computing the variations in focus measures, we can determine the degree of focusing.

**Figure 9.**
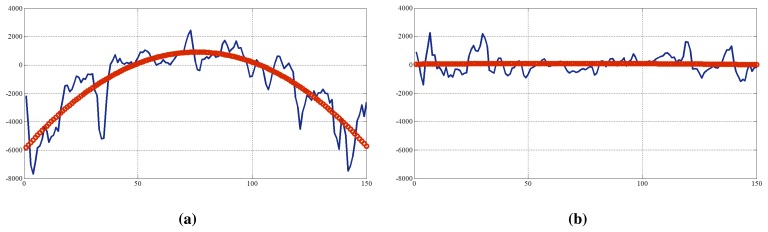
Cumulative sums of the differences (DiF) of (**a**) a real face and (**b**) a fake face.

In order to maximize the LAPM difference between regions of the nose and ears, we subtract LAPM*_E_* from LAPM*_N_* (= LAPM*_N_* – LAPM*_E_*). To analyze the differences in LAPMs (DiF, difference in focus measures) in a single dimension, we add all of the DiF in the same column. In Figure 9, blue lines describe the cumulative sums of the DiF of real and fake faces.

However, these distributions are not appropriate to be used for liveness detection without any refinement. The existence of noise affects the results. Therefore, curve fitting is performed to extract meaningful features. The sum of the DiF of real faces has a similar shape to the curvature of a quadratic equation, *y* = *ax^2^* + *bx* + *c*. In the quadratic equation, there are three coefficients, **A** = [*a b c*]*^T^*, and these are exploited as a feature for classification. To calculate the values of these coefficients, we perform error minimization [32].

**Figure 10.**
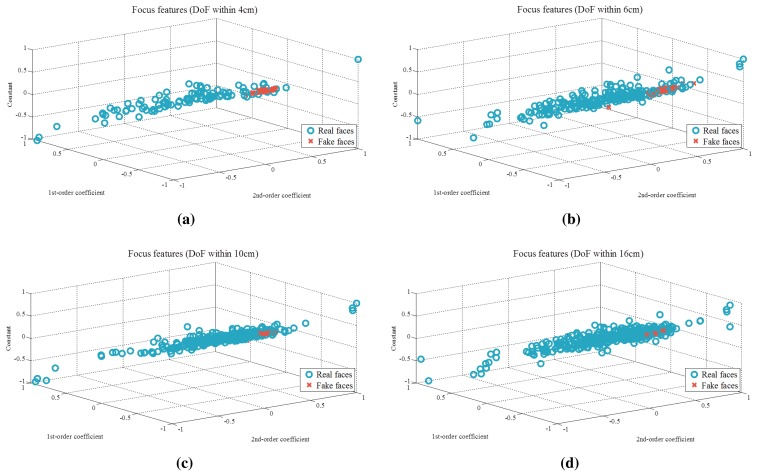
Distributions of focus features (DoF (**a**) within 4 cm, (**b**) within 6 cm, (**c**) within 10 cm and (**d**) within 16 cm).

Figure 9 presents the results of curve fitting (red circles). The curve for the cumulative sum of DiF of the real face is convex, as shown in Figure 9a, while that of the fake face is flat. In Figure 10, coefficients of quadratic equations are plotted. Blue circles are features of real faces, and red crosses are those of spoofing faces. Depending on the range of DoF, the degree of feature overlap will change.

#### 3.2.2. Power Histogram Feature

**Figure 11.**
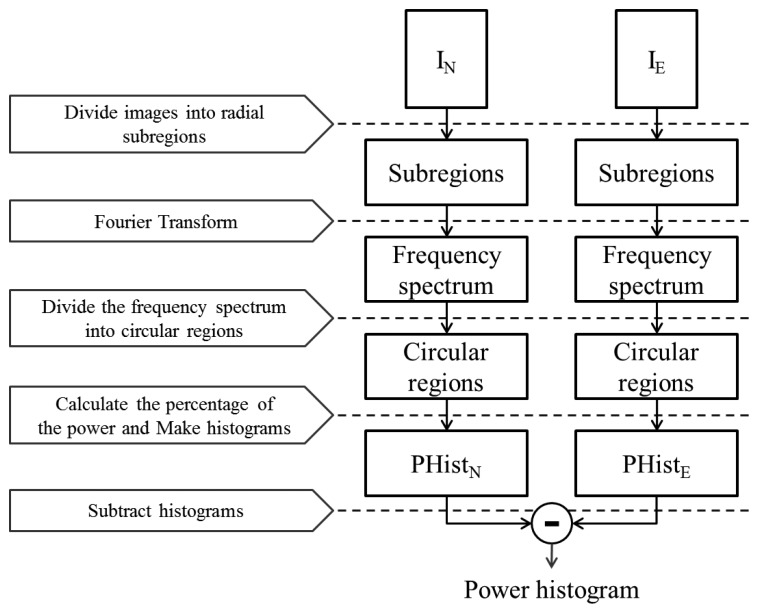
Flowchart of power histogram feature extraction.

Out-of-focus images have few edge components because the blurring filter eradicates the boundary. This affects the frequency characteristics of such images. We analyze this feature to identify forged faces. In this section, we introduce another feature, the power histogram feature, which contains spatial frequency information. The process of extracting this feature is presented in Figure 11.

**Figure 12.**
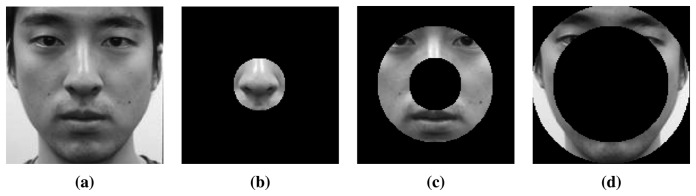
Subregions before extracting the power histogram ((**a**) original image, (**b**) Subregion 1 (subR1), (**c**) subR2 and (**d**) subR3).

In the first step, we divide a normalized image into three subregions, as shown in Figure 12. When a picture is taken focusing on the ears, we adjust the focal plane to include the ear area. Not only ears, but other components in the DoF are in focus. To analyze those components, we divide the images radially. The first subregion (subR1, Figure 12b) is the nose area, the second subregion (subR2, Figure 12c) includes the eyes and mouth, and the third subregion (subR3, Figure 12d) contains the ears and the contour of the chin.

**Figure 13.**
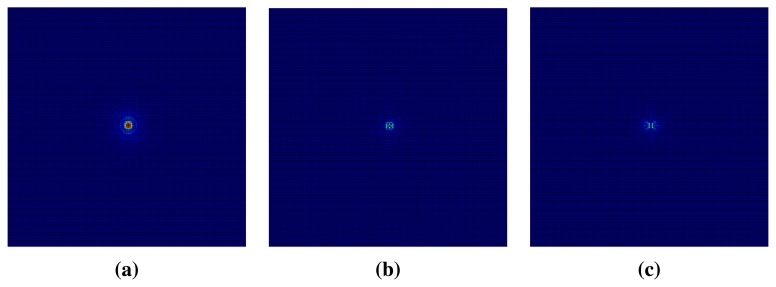
Fourier spectrums of (**a**) subR1, (**b**) subR2 and (**c**) subR3.

Using a Fourier transform, we convert subregions from the spatial domain to the frequency domain. Figure 13 illustrates center-shifted Fourier spectrums of the three described subregions with power being concentrated at the center of each spectrum. According to the subregion, the distributions of power are different. In order to analyze those distributions, we calculate the percentage of power in circular regions. We divide the frequency spectrum into several circles by allowing it to be superimposed. The percentage of power within a circular region is computed by Equation (1) [36],

(1) $$\begin{array}{c} \alpha \left(\%\right)=100\times \left[\frac{1}{P_{T}}\sum_{u,v\in C}P\left(u,v\right)\right]\\ P_{T}=\sum_{u=1}^{U}\sum_{v=1}^{V}P\left(u,v\right)\\ P\left(u,v\right)=\text{real}{\left(u,v\right)}^{2}+\text{imag}{\left(u,v\right)}^{2} \end{array}$$

where *C* is a circular region and *real*(*u*, *v*) and *imag*(*u*, *v*) are the real and imaginary parts of the frequency component, respectively. Each spectrum has a histogram, and the value of each bin is the percentage of power in each circular area. By concatenating three histograms, we can obtain a combined histogram from one image. The dimensionality of the histogram is determined by the radii of the circular regions in the frequency spectrum. With real faces, power histograms vary depending on the focus area. However, those of fake faces do not vary. We use the differences in the power histograms as a feature for liveness detection.

#### 3.2.3. GLOH Feature

**Figure 14.**
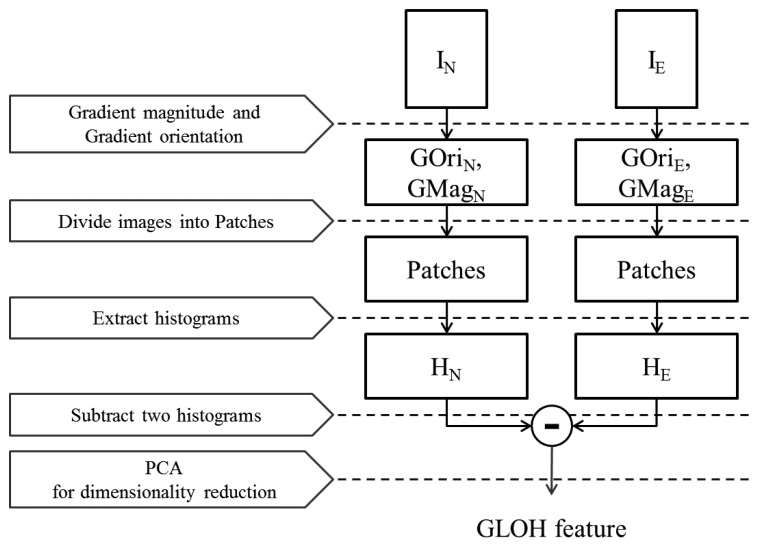
Flowchart of gradient location and orientation histogram (GLOH) feature extraction.

We extract another feature descriptor, the gradient location and orientation histogram (GLOH) [35], which is an extended version of scale-invariant feature transform (SIFT) [37] and makes it possible to consider more spatial regions, as well as making feature descriptors robust and distinctive. In this paper, we modify and apply this feature locally. Figure 14 shows the flowchart of extracting the GLOH feature.

For each Gaussian smoothed image, the gradient magnitude, *GMag*, and orientation, *GOri*, are computed by Equation (2).

(2) $$\begin{array}{c} \text{GOri}\left(x,y\right)={\text{tan}}^{-1}\frac{I\left(x,y+1\right)-I\left(x,y-1\right)}{I\left(x+1,y\right)-I\left(x-1,y\right)}\\ \text{GMag}\left(x,y\right)=\sqrt{{\left(I\left(x+1,y\right)-I\left(x-1,y\right)\right)}^{2}+{\left(I\left(x,y+1\right)-I\left(x,y-1\right)\right)}^{2}} \end{array}$$

**Figure 15.**
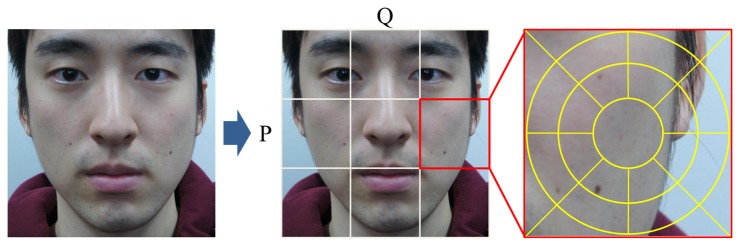
Patches in an image and polar location grids in a patch (patch size: 50 × 50).

**Figure 16.**
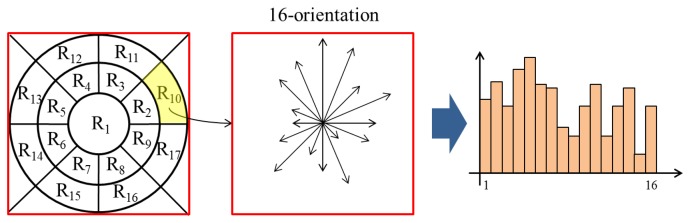
A histogram from one radial subregion.

**Figure 17.**
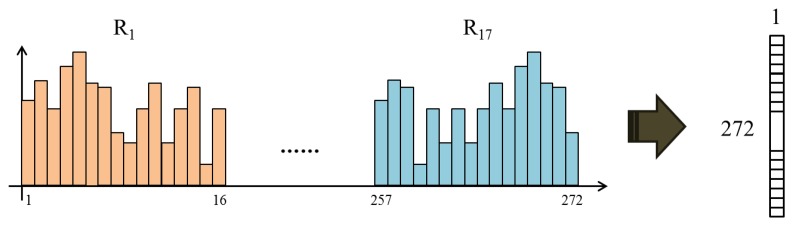
A histogram from one image patch.

Next, we divide the image into P × Q patches in order to draw features locally. Figure 15 shows how to separate the image into patches. GLOH descriptors are derived from polar location grids in patches. As shown in Figure 15, each patch is divided into 17 subregions (three bins in each radial direction and eight bins in each angular direction). Note that the central subregion is not split. In a subregion, the gradient orientations are quantized into 16 bins (Figure 16). From one patch, 17 histograms are created. We reshape these histograms into one column vector, whose dimensionality is 272 (=17 × 16), as illustrated in Figure 17. Finally, a 272 × P × Q-dimensional column vector is extracted from P × Q patches. From I*_N_* and I*_E_*, two vectors, H*_N_* and H*_E_*, are acquired, and the difference between them is determined (H*_N_* – H*_E_*). Principal component analysis (PCA) is applied to reduce the final dimensionality.

### 3.3. Classification

**Figure 18.**
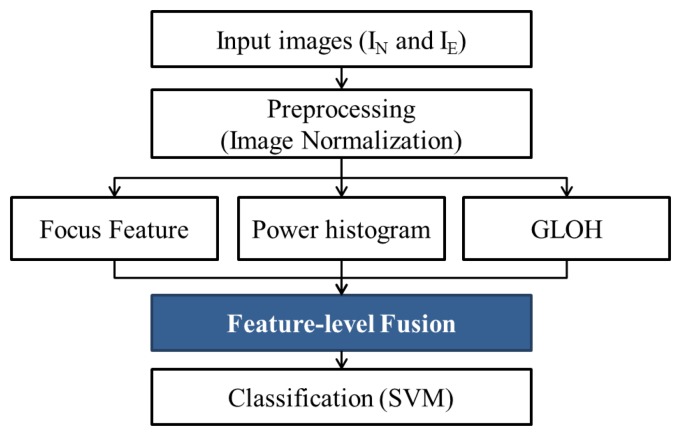
Flowchart of the feature-level fusion approach.

For classification, the support vector machine-radial basis function (SVM-RBF) is used [38]. The SVM classifier learns normalized focus, power histogram and GLOH features. Furthermore, we carry out fusion-based experiments by concatenating normalized features. Figure 18 shows the flowchart of the feature-level fusion approach. Depending on the training data and the development data, the parameter of the SVM classifier is determined.

## 4. Experimentation

Before evaluating the performances of our approaches, we collected frontal facial images from 24 subjects, because there is no open facial database that has various focusing areas. Although there are some databases for liveness detection, they do not satisfy our requirements. Therefore, we created two databases, one composed of images taken by a mirrorless camera (SONY-NEX5) and the other containing images taken by a webcam (Microsoft LifeCam Studio). The difference between the two cameras is the possibility of the accurate and delicate control of focus. With the mirrorless camera, it is possible to focus precisely on the nose or ear area. However, the webcam makes it difficult to adjust focus in detail, and users are not able to determine what is in focus. We will explain the processes of acquiring databases in the next section. We printed photos for fake faces with a Fuji Zerox ApeosPort-II C5400 printer.

For evaluations, the following measures are used.

- False acceptance rate (FAR): the proportion of fake images misclassified as real.
- False rejection rate (FRR): the proportion of real images misclassified as fake.
- Total error rate (TER): the sum of FAR and FRR. *TER* = *FAR* + *FRR*
- Half total error rate (HTER): half of the TER. *HTER* = *TER*/2

The performance of the proposed method is evaluated with our own databases. Databases are randomly categorized into 3 groups: training, development and testing sets.

- Training set (30%): to be used for training the classifier.
- Development set (30%): to be used for estimating the threshold of the classifier.
- Testing set (40%): to be used for evaluating the performance.

Thirty percent of the subjects are used for training and development, and forty percent of the subjects are used for testing. Three groups are disjoint. That is, if images of subject ‘*A*’ are used for training, they cannot be utilized for development or testing.

### 4.1. Experiment 1: Using the Mirrorless Camera Database

#### 4.1.1. Data Acquisition

**Figure 19.**
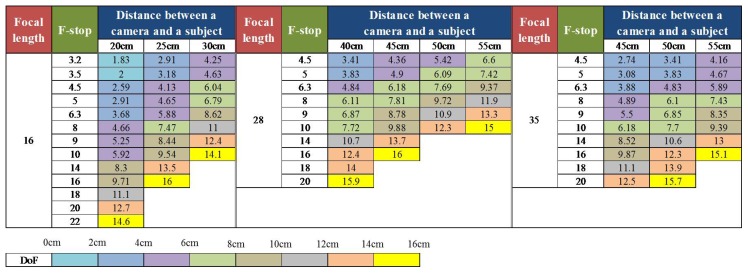
The ranges of the distance between the camera and the subject, focal length, F-stop and DoF.

With the mirrorless camera, the nose and ear areas are able to be in focus, and the DoF is manually controlled. In order to obtain images with various DoFs, we adjusted the distance between the camera and the subject, focal length and F-stop. Figure 19 shows the ranges of the parameters. Focal lengths are 16, 28 and 35 mm, and values of F-stop are changed according to the focal lengths from f/3.2 to f/22. Distances between the camera and the subject vary from 20 cm to 55 cm.

The total number of images in the mirrorless camera database is 5968 (1492 pairs of real images and 1492 pairs of fake images). The images are categorized into four groups according to the range of DoF and are listed in Table 1. The number of males is 17 and that of females is 7.

**Figure 20.**
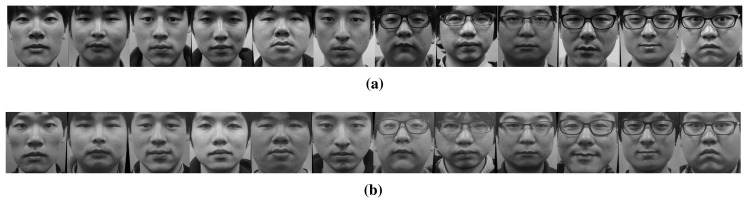
Normalized images of (**a**) real and (**b**) fake faces.

The size of each normalized image is 150 by 150 pixels, and the distance between the eyes is 70 pixels. Figure 20 shows real (a) and fake (b) samples from the database.

#### 4.1.2. Experimental Results

We carry out experiments in accordance with the types of features, and the detailed results are described in Appendix A. The following shows the performance of the concatenated features. The process of combining features is carried out in the feature level. For high performance, we choose features based on the above results. Modified Laplacian (LAPM) and wavelet sum (WAVS) are used as focus features. In the case of the power histogram feature, the radii of the circular regions are 5, 15, 30, 50 and 75. GLOH features are extracted using 75 × 75 patches without allowing overlap. In order to reduce the dimensionality of the GLOH features, we apply PCA and use several eigenvectors whose variances are 90%. Table 2 shows the denotations of the features.

**Figure 21.**
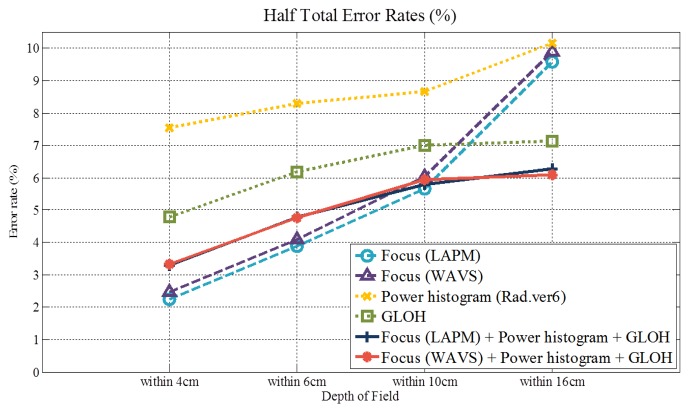
HTERs (%) of features.

**Figure 22.**
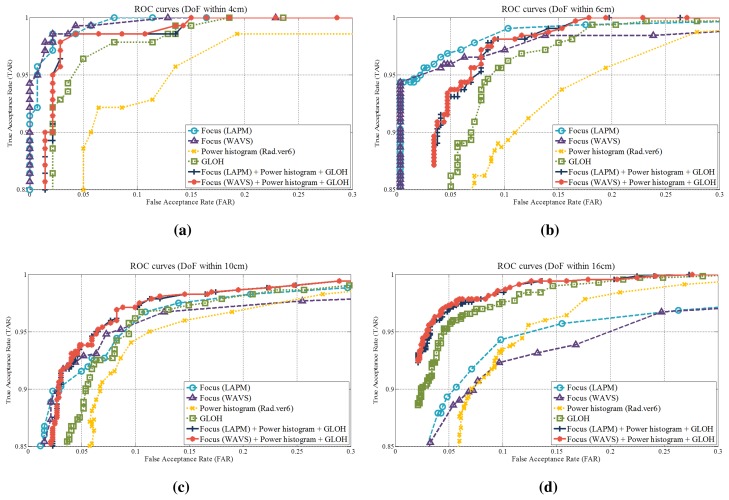
ROC curves of the feature-level fusion (DoF (**a**) within 4 cm, (**b**) within 6 cm, (**c**) within 10 cm and (**d**) within 16 cm).

Table 3 and Figures 21 and 22 illustrate the results of the fusion-based methods. When the DoF is shallow (within 4 cm and 6 cm), the performances of focus features (LAPM and WAVS) are better than those of other features. However, as the DoF becomes deeper, the performances of focus features deteriorate. In the case of the GLOH and fusion-based features, the performances are maintained compared to other features. Especially, the HTERs of the fusion-based features under 16-cm DoF are lower than those of other features (6.27% and 6.08%). These numerical results demonstrate that the fusion-based methods are prominent when the effect of defocusing is low.

### 4.2. Experiment 2: Using the Webcam Database

#### 4.2.1. Data Acquisition

For evaluations, we gathered facial data using Microsoft LifeCam Studio. Using the provided program, we could control the lens motor step from 0 to 40. Therefore, one input sequence is composed of 41 images. Among those, we choose I*_N_* and I*_E_*, as mentioned in Section 3.1.2. The distance between the webcam and the subject is about 20 cm, so that the image can contain a whole face. The number of real face sequences is 94. Normal prints and an HD tablet (iPad 2) are used as spoofing attacks, and the number of sequences is 240 and 120 respectively. Five-fold cross-validation is applied for the evaluation.

#### 4.2.2. Experimental Results

Numerical results are listed in Table 4. The good performance is maintained, even though the webcam database cannot express depth information well compared to the mirrorless camera database. The results of the combined features are the best, and the HTERs of them are 3.02% under normal print attack and 3.15% under HD tablet attack. These experiments show the possibility that our proposed method can be used in security systems at a low cost and with low specification devices. Furthermore, if detailed adjustment of the focus is possible in the device, our method can improve the performance more.

### 4.3. Discussion

Due to the characteristic of our proposed method, it is impossible to apply our method to open databases, such as the CASIA database [39] and the Replay-Attack database [40]. Therefore, we conducted comparative experiments by applying other methods to our own database. Table 5 demonstrates the performance comparison between our proposed method and other methods.

Other methods [9,41,42] detect the liveness based on textural analysis (local binary patterns) or frequency components (difference of Gaussian, power spectrum). Even though they have an advantage in terms of using a single image, the performances for our own database are not remarkable, regardless of the DoF; whereas the previous work [32] shows a good result relatively at the within 4-cm DoF. However, when the DoF is deep, the performance of [32] deteriorates. This represents that the performance of the previous system is determined depending on the method of input picture collection with great effects on defocus.

In order to overcome this limitation, we propose our system by considering two factors. The first is by supplementing features. By adding other feature descriptors, we try to maintain good performance, even though the DoF becomes deeper. In the case of the GLOH feature [35], it has high matching scores for images with severe blur, whereas local features used in other methods [9,41,42] are not proper for the defocused images to be compared to the GLOH feature descriptor. The influence of the GLOH feature can be confirmed in the previous Section 4.1.2. In Figure 21, the performances of the focus and power histogram features are deteriorated in accordance with the increase of the DoF. However, the performance of the GLOH feature is maintained. As a result, we can achieve 6.51% HTER (feature-fusion) at a DoF within 16 cm by using additional features specialized for the defocused images. These results are better than the HTERs of other methods and the previous method [32], which uses only a focus feature of 20.8%.

The second way to mitigate the weakness of the previous study [32] is the use of the webcam database. Digital cameras, such as DSLR and mirrorless cameras, have high specifications and make it possible to manually adjust the DoF and focusing areas. However, due to their high cost, people might be unwilling to use digital cameras for image acquisition in anti-spoofing algorithms. Webcams are cheaper than digital cameras and are utilized broadly. With the webcam, we created a database and conducted experiments. As a result, we accomplish 3.02% HTER with the combined feature. The performance with the webcam database is similar to that with the mirrorless camera database.

Even though we show the good performance for liveness detection, our method has a disadvantage in the process of acquiring and normalizing images. In this paper, we set the focus on the ears and nose and find the centers of the eyes manually. In order to apply our proposed method to the security systems at low cost and with low specification devices, like smartphones, facial components must be detected automatically. Recently, many studies for feature point extraction have been in progress, and most cameras and smartphones have a face priority auto focusing function [43–45], which helps to obtain face-focused images by automatically controlling the lens actuator. If these technologies are utilized, the limitation of our method will be settled and applicable to the devices. Moreover, it will strengthen the security of smartphones.

## 5. Conclusion and Future Work

We proposed a face liveness detection method based on the characteristics of defocus. Our method pays attention to the difference between the properties of real and 2D fake faces. We use focus, power histogram and GLOH as feature descriptors and classify spoofing faces in terms of the feature-level fusion processes. Our experimental results show 3.29% HTER when the DoF of images is within 4 cm. Moreover, by applying various features, we overcome the limitation of DoF without adding any other sensors. Furthermore, through experiments with a webcam, we confirm that the good performance of our method is maintained.

Even though our proposed method yields good results, it has a limitation for being applied to camera-embedded security systems, such as smartphones, because of the manual processes to acquire the focused images and to detect facial components. Therefore, in future work, we will improve our method in order for it to operate automatically in the image acquisition and preprocessing and to make it possible to embed our method on a smart devices. Furthermore, we will consider more robust countermeasures against videos and 3D attacks by analyzing textural and temporal characteristics. Furthermore, we will advance our method using a light-field camera, which can acquire various focusing information in the spatial domain using a microlens array.

## Figures and Tables

**Table 1.** The number of pairs of images in the database.

**Depth of Field**	**within 4 cm**	**within 6 cm**	**within 10 cm**	**within 16 cm**
Real	336	767	1149	1492
Fake	336	767	1149	1492

**Table 2.** Denotations.

**Denotation**	**Specification**
Focus (LAPM)	Modified Laplacian
Focus (WAVS)	Wavelet sum
Power hist	Rad.ver6 (radii = 5, 15, 30, 50, 75)
GLOH	Patch75, No overlapping, PCA 90%
Fusion.ver1	Focus (LAPM) + Power hist + GLOH
Fusion.ver2	Focus (WAVS) + Power hist + GLOH

**Table 3.** Half total error rates (HTERs) (%) of the experiments with the mirrorless camera database.

	**within 4 cm**			**within 6 cm**			**within 10 cm**			**within 16 cm**		

	**Dev (mean** ± **std)**	**Test (mean** ± **std)**	**sigma**	**Dev (mean** ± **std)**	**Test (mean** ± **std)**	**sigma**	**Dev (mean** ± **std)**	**Test (mean** ± **std)**	**sigma**	**Dev (mean** ± **std)**	**Test (mean** ± **std)**	**sigma**

Focus (LAPM)	0.92 ± 0.40	**2.25** ± **1.54**	0.09	2.72 ± 0.89	**3.89** ± **0.70**	0.09	5.49 ± 0.95	**5.66** ± **0.53**	0.08	7.59 ± 1.43	9.57 ± 1.84	0.08
Focus (WAVS)	1.28 ± 0.97	**2.46** ± **0.91**	0.15	3.37 ± 1.32	**4.09** ± **0.69**	0.095	5.87 ± 0.57	6.04 ± 0.66	0.10	8.33 ± 1.40	9.90 ± 1.20	0.09
Power hist	5.15 ± 1.76	7.54 ± 1.18	0.90	6.50 ± 1.62	8.29 ± 1.42	1.00	6.98 ± 1.13	8.67 ± 1.34	0.70	9.29 ± 1.58	10.1 ± 1.96	0.75
GLOH	3.52 ± 1.24	4.79 ± 1.37	1.75	5.09 ± 0.88	6.19 ± 0.87	3.00	5.08 ± 0.88	7.00 ± 0.99	2.90	6.98 ± 1.26	**7.13** ± **1.31**	3.55
Fusion.ver1	2.45 ± 1.27	**3.29** ± **1.44**	2.30	4.06 ± 0.90	4.78 ± 1.08	3.40	3.92 ± 0.89	**5.79** ± **1.05**	3.65	5.58 ± 1.66	**6.27** ± **1.80**	4.55
Fusion.ver2	2.45 ± 1.27	3.32 ± 1.42	2.40	4.06 ± 0.89	**4.76** ± **0.86**	4.50	3.91 ± 0.96	**5.93** ± **1.23**	4.15	5.57 ± 1.62	**6.08** ± **1.72**	5.25

**Table 4.** HTERs (%) of experiments with the webcam database.

	**Normal Print**	**HD Tablet**
	**(mean** ± **std)**	**(mean** ± **std)**
Focus (LAPM)	8.29 ± 0.45	10.0 ± 0.45
Focus (WAVS)	6.48 ± 0.36	7.54 ± 0.49
Power hist	7.93 ± 0.45	7.25 ± 0.43
GLOH	6.09 ± 0.55	5.28 ± 1.06
Fusion.ver1	**3.39 ± 0.46**	**3.30 ± 0.66**
Fusion.ver2	**3.02 ± 0.47**	**3.15 ± 0.45**

**Table 5.** Performance comparison (HTER (%)).

	**within 4 cm**			**within 6 cm**			**within 10 cm**			**within 16 cm**		

	**Dev (mean** ± **std)**	**Test (mean** ± **std)**	**sigma**	**Dev (mean** ± **std)**	**Test (mean** ± **std)**	**sigma**	**Dev (mean** ± **std)**	**Test (mean** ± **std)**	**sigma**	**Dev (mean** ± **std)**	**Test (mean** ± **std)**	**sigma**

Zhang [41]	29.2 ± 4.12	39.2 ± 3.55	2.00	26.9 ± 4.45	33.9 ± 4.00	2.40	29.3 ± 2.99	35.4 ± 5.70	2.95	25.7 ± 4.70	36.9 ± 4.41	3.85
Kim [9]	12.6 ± 2.86	17.2 ± 3.70	15.0	18.8 ± 5.74	18.8 ± 5.55	18.5	17.65 ± 3.35	20.4 ± 4.05	17.3	23.9 ± 5.28	18.9 ± 6.03	17.2
Määttä [42]	19.7 ± 7.11	22.2 ± 4.35	-	20.4 ± 5.64	21.6 ± 2.52	-	24.4 ± 4.54	23.1 ± 5.40	-	22.6 ± 6.99	21.0 ± 3.14	-
Kim [32]	9.34 ± 4.07	8.39 ± 2.63	-	11.9 ± 1.90	12.1 ± 1.59	-	15.9 ± 1.97	16.3 ± 2.27	-	19.1 ± 2.05	20.8 ± 2.10	-
Fusion.ver1	2.45 ± 1.31	3.39 ± 1.46	2.30	4.51 ± 1.04	4.87 ± 1.13	3.45	4.07 ± 1.03	5.60 ± 0.83	3.45	5.19 ± 1.60	6.71 ± 1.65	4.55
Fusion.ver2	2.50 ± 1.33	3.39 ± 1.45	2.35	4.55 ± 0.92	4.80 ± 0.87	3.65	4.09 ± 1.07	5.82 ± 1.13	4.05	5.20 ± 1.57	6.51 ± 1.57	5.25

**Figure A1.**
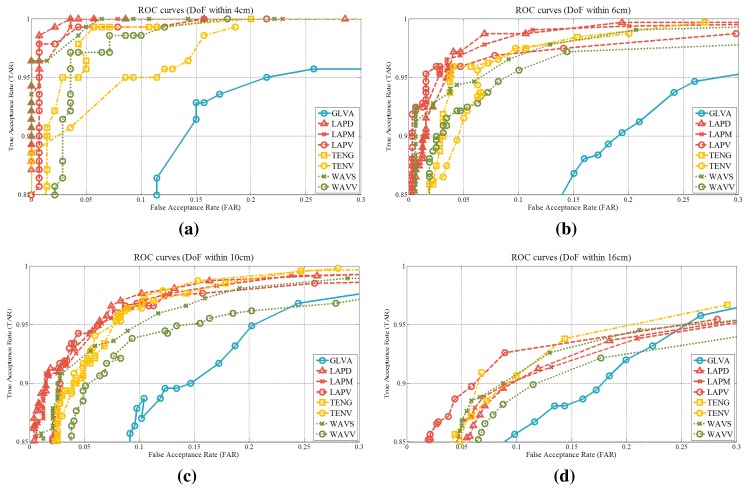
ROC curves of the focus features (DoF (**a**) within 4 cm, (**b**) within 6 cm, (**c**) within 10 cm and (**d**) within 16 cm).

**Figure A2.**
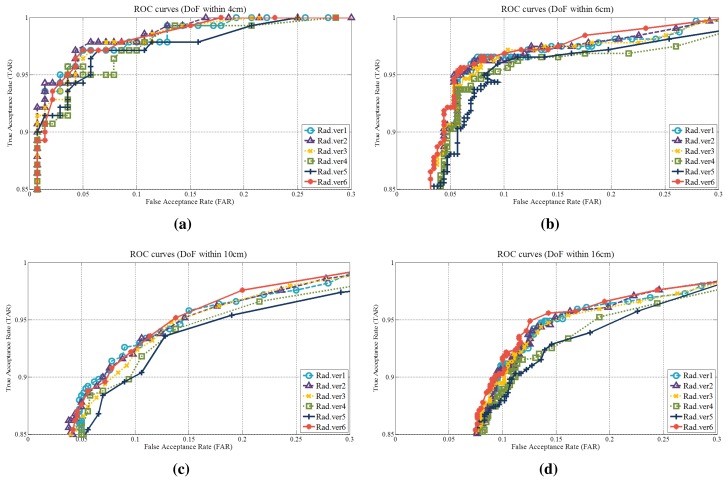
ROC curves of the power histogram features (DoF (**a**) within 4 cm, (**b**) within 6 cm, (**c**) within 10 cm and (**d**) within 16 cm).

**Figure A3.**
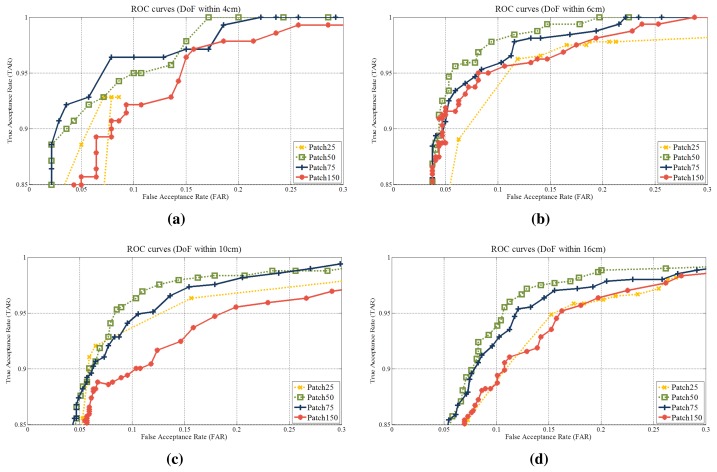

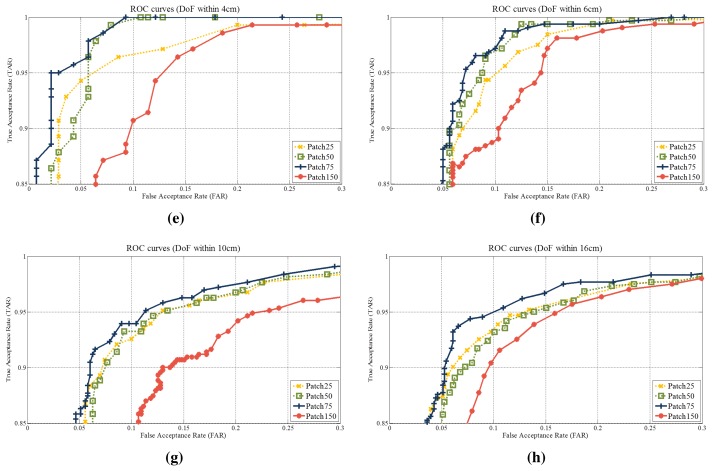
ROC curves of the GLOH features (overlap: PCA 90%, DoF (**a**) within 4 cm, (**b**) within 6 cm, (**c**) within 10 cm and (**d**) within 16 cm; and NO overlap: PCA 90%, DoF (**e**) within 4 cm, (**f**) within 6 cm, (**g**) within 10 cm and (**h**) within 16 cm).

## A. Appendix

### Experiments According to the Type of Features

We carry out experiments in accordance with the types of features. The following shows the performance of our proposed methods.

#### A.1. Focus Feature

We conduct experiments with eight types of focus features. Eight focus features are categorized into four groups: statistic-based, Laplacian-based, gradient-based and wavelet-based operators [4]. The focus features are listed in Table A1. Related equations are organized in [4].

Table A2 and Figure A1 show HTERs and receiver operating characteristic (ROC) curves of focus features according to the range of the DoF. In general, the performance of the Laplacian group is better than those of other groups. As depicted in Figure A1, focus features in the Laplacian group swarm in the upper side. Especially, modified Laplacian (LAPM) has stable and prominent results all over the DoF (1.64% HTER under the within 4-cm DoF and 8.93% HTER under the within 16-cm DoF). The sum of wavelet coefficients (WAVS) also shows good performance. When the DoF is shallow, the effect of defocusing is great. This makes the focus features of real and fake faces more discriminative. As a result, focus features, except gray-level variance (GLVA), yield the best performances under the within 4-cm DoF. The GLVA focus feature, unusually, has the best performance when the DoF is within 16 cm (12.4% HTER). GLVA is the simple variance of the gray-scale image. Compared to other focus features, it is inadequate to represent the difference between the focused and defocused regions regardless of the DoF.

**Table A1.** Focus features.

**Focus Feature (Abbreviation)**	**Focus Feature (Full Name)**	**Category**
GLVA	Gray-level variance	Statistic
LAPD	Diagonal Laplacian	Laplacian
LAPM	Modified Laplacian	Laplacian
LAPV	Variance of Laplacian	Laplacian
TENG	Tenengrad	Gradient
TENV	Tenengrad variance	Gradient
WAVS	Sum of wavelet coefficients	Wavelet
WAVV	Variance of wavelet coefficients	Wavelet

**Table A2.** HTERs (%) of the focus features.

	**within 4 cm**			**within 6 cm**			**within 10 cm**			**within 16 cm**		

	**Dev (mean** ± **std)**	**Test (mean** ± **std)**	**sigma**	**Dev (mean** ± **std)**	**Test (mean** ± **std)**	**sigma**	**Dev (mean** ± **std)**	**Test (mean** ± **std)**	**sigma**	**Dev (mean** ± **std)**	**Test (mean** ± **std)**	**sigma**

GLVA	14.4 ± 2.77	16.6 ± 2.41	0.3	14.1 ± 2.30	13.6 ± 2.20	0.09	11.4 ± 2.34	12.5 ± 1.15	0.08	10.7 ± 2.01	12.4 ± 1.59	0.1
LAPD	1.02 ± 1.15	**1.82** ± **0.59**	0.15	3.46 ± 1.18	3.97 ± 1.09	0.20	4.91 ± 0.81	**5.75** ± **0.56**	0.135	8.61 ± 1.78	9.41 ± 1.64	0.09
LAPM	1.07 ± 1.16	**1.64** ± **0.59**	0.2	3.08 ± 0.99	**3.57** ± **1.05**	0.09	4.97 ± 0.88	**6.10** ± **0.39**	0.085	8.56 ± 1.40	**8.93** ± **1.90**	0.06
LAPV	1.22 ± 0.80	2.50 ± 2.61	0.35	3.06 ± 0.92	**3.16** ± **0.86**	0.075	5.67 ± 1.05	6.36 ± 0.63	0.25	8.53 ± 1.51	**8.48** ± **1.31**	0.07
TENG	3.72 ± 1.91	4.68 ± 1.86	0.09	4.27 ± 0.98	4.88 ± 0.55	0.065	5.59 ± 1.07	6.17 ± 1.05	0.065	8.05 ± 1.69	**9.14** ± **1.30**	0.055
TENV	4.94 ± 1.89	7.11 ± 3.34	0.135	5.41 ± 1.40	6.79 ± 2.03	0.03	7.53 ± 1.25	8.44 ± 1.37	0.06	9.22 ± 2.91	10.8 ± 1.78	0.01
WAVS	1.22 ± 1.11	**2.00** ± **0.89**	0.3	3.53 ± 1.34	**3.50** ± **0.91**	0.2	5.53 ± 0.69	**6.15** ± **0.72**	0.145	8.83 ± 1.73	9.79 ± 1.05	0.08
WAVV	2.65 ± 1.44	4.57 ± 2.44	0.55	5.70 ± 0.84	5.66 ± 1.32	0.09	8.21 ± 0.85	8.96 ± 1.44	0.08	10.7 ± 1.86	11.4 ± 1.73	0.45

#### A.2. Power Histogram Feature

In order to find that how to divide the frequency spectrum that yields good performance, we carry out experiments taking the radii of circular regions in Table A3. The dimensionality is the length of the concatenated histograms of the three subregions.

**Table A3.** Radii of circular regions.

**Version**	**Radii**	**Dimensionality**
Rad.ver1	[1:1:75]	225
Rad.ver2	[3:3:75]	75
Rad.ver3	[5:5:75]	45
Rad.ver4	[10:10:75]	21
Rad.ver5	[15:15:75]	15
Rad.ver6	[5 15 30 50 75]	15

Table A4 describes the numerical results, and Figure A2 illustrates the distributions of the HTERs and ROC curves. When the averages of HTERs are calculated respectively, Rad.ver6 shows a good performance: 7.69% HTER. Even though the dimensionality of the power histogram feature is low, it yields the best performance compared to the others.

**Table A4.** HTERs (%) of the power histogram features.

	**within 4 cm**			**within 6 cm**			**within 10 cm**			**within 16 cm**			**Avg.**

	**Dev (mean** ± **std)**	**Test (mean** ± **std)**	**sigma**	**Dev (mean** ± **std)**	**Test (mean** ± **std)**	**sigma**	**Dev (mean** ± **std)**	**Test (mean** ± **std)**	**sigma**	**Dev (mean** ± **std)**	**Test (mean** ± **std)**	**sigma**	

Rad.ver1	5.61 ± 1.73	**7.04** ± **1.86**	2.65	7.76 ± 2.24	**8.19** ± **1.01**	3.05	7.40 ± 1.64	**8.34** ± **1.19**	2.95	8.30 ± 2.13	**10.1** ± **2.04**	3.50	7.84
Rad.ver2	4.64 ± 1.55	**6.93** ± **2.20**	2.60	6.98 ± 1.46	**7.43** ± **1.70**	2.20	7.85 ± 1.51	**8.42** ± **1.29**	1.45	8.54 ± 1.85	11.5 ± 1.93	1.55	7.79
Rad.ver3	6.33 ± 2.57	**7.04** ± **1.77**	1.85	7.42 ± 1.51	**8.99** ± **3.32**	1.15	7.30 ± 0.94	9.32 ± 0.96	1.25	8.74 ± 1.84	**10.2** ± **1.22**	1.00	8.17
Rad.ver4	5.10 ± 0.96	7.36 ± 2.17	1.05	7.04 ± 1.80	9.71 ± 1.61	0.90	7.50 ± 1.55	9.63 ± 1.71	1.50	9.21 ± 1.75	11.3 ± 0.83	0.65	8.36
Rad.ver5	6.12 ± 2.44	7.36 ± 1.54	1.40	8.84 ± 1.51	9.88 ± 2.81	0.75	8.05 ± 0.96	9.37 ± 0.97	1.00	9.67 ± 1.61	10.9 ± 1.46	0.50	8.77
Rad.ver6	5.15 ± 1.90	7.07 ± 1.26	1.50	5.88 ± 1.22	9.15 ± 2.41	1.55	6.99 ± 0.76	**8.85** ± **1.92**	1.30	8.50 ± 1.12	**9.93** ± **1.02**	0.90	**7.69**

#### A.3. GLOH Feature

We perform experiments by altering the size of the patch, the energy percentage in PCA and whether the patches are overlapped or not. In Tables A5, A6, A7, A8, A9 and A10, numerical results are listed, and Figure A3 describes the ROC curves.

As shown in Figure A3, the performances with and without allowing the overlap of patches are similar. In terms of the computational cost, the overlap of patches is not effective. Therefore, it is better not to overlap the patches to extract the GLOH features. In the case of the energy percentage in PCA, when the percentage is 98%, the performance is worse than those under 90% and 95%.

Additionally, experiments are carried out depending on the size of the patch. When the GLOH features are extracted from the whole image, the performance is the worst, and those features cannot represent the spatial properties sufficiently. As the size of the patch is 75 × 75 and the energy percentage in PCA is 90% without allowing the overlap, the performance is the best (7.75% HTER under the within 16-cm DoF).

**Table A5.** HTERs (%) of GLOH features (overlap, PCA 90%).

	**within 4 cm**			**within 6 cm**			**within 10 cm**			**within 16 cm**		

	**Dev (mean** ± **std)**	**Test (mean** ± **std)**	**sigma**	**Dev (mean** ± **std)**	**Test (mean** ± **std)**	**sigma**	**Dev (mean** ± **std)**	**Test (mean** ± **std)**	**sigma**	**Dev (mean** ± **std)**	**Test (mean** ± **std)**	**sigma**

Patch25	6.17 ± 1.33	7.61 ± 1.67	0.65	7.42 ± 1.19	8.86 ± 1.11	1.40	7.52 ± 1.64	8.14 ± 1.77	1.70	8.52 ± 1.36	8.92 ± 1.49	2.25
Patch50	4.85 ± 1.62	**7.36** ± **2.21**	1.55	6.74 ± 1.21	**6.47** ± **0.67**	2.30	6.32 ± 1.20	**6.70** ± **0.82**	2.80	6.83 ± 1.51	**8.20** ± **1.72**	3.30
Patch75	5.20 ± 1.65	7.64 ± 1.38	1.80	6.23 ± 0.80	7.01 ± 0.71	2.70	6.58 ± 1.39	6.87 ± 0.78	3.10	6.93 ± 1.20	8.25 ± 0.96	4.20
Patch150	6.48 ± 2.20	9.57 ± 1.86	2.95	8.42 ± 1.07	8.82 ± 2.05	3.70	7.42 ± 1.17	9.36 ± 0.86	3.20	8.83 ± 1.74	10.1 ± 1.52	3.40

**Table A6.** HTERs (%) of GLOH features (overlap, PCA 95%).

	**within 4 cm**			**within 6 cm**			**within 10 cm**			**within 16 cm**		

	**Dev (mean** ± **std)**	**Test (mean** ± **std)**	**sigma**	**Dev (mean** ± **std)**	**Test (mean** ± **std)**	**sigma**	**Dev (mean** ± **std)**	**Test (mean** ± **std)**	**sigma**	**Dev (mean** ± **std)**	**Test (mean** ± **std)**	**sigma**

Patch25	8.57 ± 1.68	8.64 ± 2.60	0.70	10.0 ± 1.86	9.05 ± 1.12	1.55	10.8 ± 6.04	10.8 ± 4.19	2.25	8.90 ± 1.47	10.3 ± 1.26	2.60
Patch50	6.07 ± 2.97	**7.89** ± **1.81**	1.40	6.50 ± 1.10	**7.49** ± **0.76**	2.50	6.26 ± 1.31	**7.36** ± **1.34**	3.20	7.46 ± 1.52	**8.44** ± **0.92**	4.20
Patch75	7.24 ± 1.84	8.14 ± 1.84	2.15	7.40 ± 1.58	8.58 ± 1.03	3.35	7.99 ± 0.82	7.73 ± 1.06	4.40	7.52 ± 1.57	9.04 ± 1.91	5.90
Patch150	7.60 ± 2.38	10.0 ± 1.34	2.95	10.6 ± 2.36	9.58 ± 1.77	5.30	9.82 ± 1.74	9.67 ± 1.56	3.75	10.7 ± 2.02	10.3 ± 1.63	4.80

**Table A7.** HTERs (%) of GLOH features (overlap, PCA 98%).

	**within 4 cm**			**within 6 cm**			**within 10 cm**			**within 16 cm**		

	**Dev (mean** ± **std)**	**Test (mean** ± **std)**	**sigma**	**Dev (mean** ± **std)**	**Test (mean** ± **std)**	**sigma**	**Dev (mean** ± **std)**	**Test (mean** ± **std)**	**sigma**	**Dev (mean** ± **std)**	**Test (mean** ± **std)**	**sigma**

Patch25	19.1 ± 11.5	20.3 ± 10.2	0.90	12.5 ± 4.69	12.2 ± 4.32	1.50	9.08 ± 1.29	9.38 ± 1.45	2.45	10.2 ± 2.27	11.0 ± 2.58	3.75
Patch50	6.83 ± 2.37	**7.17** ± **2.13**	1.50	6.79 ± 1.48	**7.61** ± **0.96**	3.10	7.32 ± 0.98	**7.33** ± **1.30**	4.15	7.80 ± 1.92	**9.23** ± **1.37**	5.05
Patch75	7.39 ± 1.58	9.25 ± 2.46	2.05	7.73 ± 1.12	8.94 ± 1.44	4.20	8.43 ± 2.15	9.04 ± 1.20	6.00	8.02 ± 1.48	10.4 ± 2.23	9.55
Patch150	7.70 ± 1.62	10.3 ± 2.23	4.60	10.2 ± 2.24	11.9 ± 1.35	7.30	10.7 ± 2.15	11.7 ± 1.96	7.25	11.2 ± 1.26	12.6 ± 1.86	8.60

**Table A8.** HTERs (%) of GLOH features (no overlap, PCA 90%).

	**within 4 cm**			**within 6 cm**			**within 10 cm**			**within 16 cm**		

	**Dev (mean** ± **std)**	**Test (mean** ± **std)**	**sigma**	**Dev (mean** ± **std)**	**Test (mean** ± **std)**	**sigma**	**Dev (mean** ± **std)**	**Test (mean** ± **std)**	**sigma**	**Dev (mean** ± **std)**	**Test (mean** ± **std)**	**sigma**

Patch25	5.05 ± 1.00	**5.14** ± **1.37**	0.90	6.90 ± 1.21	7.10 ± 1.26	1.75	6.59 ± 0.82	7.60 ± 1.43	2.45	7.22 ± 1.47	8.89 ± 0.87	3.00
Patch50	4.69 ± 1.89	6.67 ± 1.54	1.55	6.89 ± 1.12	6.63 ± 1.83	2.50	6.07 ± 0.90	7.26 ± 0.68	3.15	7.53 ± 1.09	8.56 ± 1.25	3.65
Patch75	3.11 ± 1.16	5.67 ± 1.74	1.75	5.37 ± 0.83	**6.42** ± **1.73**	2.85	5.60 ± 0.65	**7.07** ± **1.20**	2.85	5.75 ± 1.62	**7.75** ± **1.13**	4.10
Patch150	6.48 ± 2.20	9.57 ± 1.86	2.95	8.42 ± 1.07	8.82 ± 2.05	3.70	7.42 ± 1.17	9.36 ± 0.86	3.20	8.83 ± 1.74	10.1 ± 1.52	3.40

**Table A9.** HTERs (%) of GLOH features (no overlap, PCA 95%).

	**within 4 cm**			**within 6 cm**			**within 10 cm**			**within 16 cm**		

	**Dev (mean** ± **std)**	**Test (mean** ± **std)**	**sigma**	**Dev (mean** ± **std)**	**Test (mean** ± **std)**	**sigma**	**Dev Test (mean** ± **std) (mean** ± **std)**		**sigma**	**Dev (mean** ± **std)**	**Test (mean** ± **std)**	**sigma**

Patch25	7.85 ± 3.94	8.21 ± 3.54	0.90	7.51 ± 1.11	8.65 ± 1.74	1.95	7.48 ± 1.21	8.35 ± 1.56	3.00	8.44 ± 1.53	9.45 ± 1.92	3.60
Patch50	6.02 ± 1.62	8.67 ± 3.59	1.50	7.49 ± 0.91	**6.73** ± **0.86**	3.35	7.04 ± 0.81	7.54 ± 1.01	5.15	9.40 ± 2.56	**7.91** ± **1.20**	5.40
Patch75	4.69 ± 1.39	**6.10** ± **1.61**	2.20	7.13 ± 2.41	7.16 ± 1.07	3.65	6.74 ± 1.54	**6.62** ± **0.86**	4.00	7.45 ± 1.96	8.52 ± 1.73	5.15
Patch150	7.60 ± 2.38	10.0 ± 1.34	2.95	10.6 ± 2.36	9.58 ± 1.77	5.30	9.82 ± 1.74	9.67 ± 1.56	3.75	10.7 ± 2.02	10.3 ± 1.63	4.80

**Table A10.** HTERs (%) of GLOH features (no overlap, PCA 98%).

	**within 4 cm**			**within 6 cm**			**within 10 cm**			**within 16 cm**		

	**Dev (mean** ± **std)**	**Test (mean** ± **std)**	**sigma**	**Dev (mean** ± **std)**	**Test (mean** ± **std)**	**sigma**	**Dev (mean** ± **std)**	**Test (mean** ± **std)**	**sigma**	**Dev (mean** ± **std)**	**Test (mean** ± **std)**	**sigma**

Patch25	11.0 ± 5.95	11.4 ± 6.54	1.20	8.10 ± 1.38	8.94 ± 1.95	2.55	7.16 ± 1.24	9.31 ± 1.69	3.60	8.90 ± 2.23	9.92 ± 1.42	4.10
Patch50	6.78 ± 1.96	7.75 ± 1.67	1.80	6.30 ± 1.22	8.88 ± 2.45	3.85	6.30 ± 1.83	**7.39** ± **1.17**	5.40	8.87 ± 2.08	**7.83** ± **1.57**	8.05
Patch75	6.17 ± 1.62	**6.57** ± **1.47**	2.30	6.97 ± 2.07	**7.57** ± **1.76**	6.25	6.90 ± 1.83	7.75 ± 1.17	6.65	9.75 ± 2.28	8.46 ± 1.20	9.60
Patch150	7.70 ± 1.62	10.3 ± 2.23	4.60	10.2 ± 2.24	11.9 ± 1.35	7.30	10.7 ± 2.15	11.7 ± 1.96	7.25	11.2 ± 1.26	12.6 ± 1.86	8.60
